# Clinical Outcome of Single-Stage Posterior Decompression and Stabilisation for Spine Metastasis

**DOI:** 10.5704/MOJ.2411.005

**Published:** 2024-11

**Authors:** CS Wang, Z Atan

**Affiliations:** Department of Orthopaedics, Hospital Raja Permaisuri Bainun, Ipoh, Malaysia

**Keywords:** spine metastases, surgical decompression, posterior spinal stabilisation, clinical outcome

## Abstract

**Introduction:**

Surgical treatment for indicated spinal metastases cases is an option to improve patients’ outcomes. Local data in analysing the potential of patients’ improvement after surgical treatment are limited. We intend to review the clinical outcomes of surgeries performed for cancer patients who were diagnosed with spinal metastases. We aim to identify factors associated with improved spinal pain, neurological deficit and patient survival.

**Material and Methods:**

The medical records of 51 patients who were diagnosed with thoracolumbar spinal metastatic tumour and underwent palliative single-stage posterior approach spinal surgery between June 2015 and June 2022 were recruited retrospectively. Patient demographic data, pre-operative and post-operative pain scores, neurological assessment and survival duration were collected from the medical records. Radiological findings were studied using respective imaging and reports.

**Results:**

The mean age was 57.5 years, and the median survival was nine months after the surgical treatment. The post-operative pain improvements were statistically significant at two weeks (VAS improved from 5 to 2), and three months follow-up VAS was one (p<0.001 and p=0.009, respectively). At initial presentation, patients with a single-level spinal involvement had higher VAS compared to multiple spinal metastases (p=0.018). A total of 18 (35.3%) patients had improved one or more ASIA grades, of which eight (15.7%) of them had gain of ambulatory function (p<0.001). Twenty-seven (52.9%) patients were ambulatory post-operative. The slow growth type of primary carcinoma, post-operative ambulatory ability, and the absence of perioperative morbidity were factors associated with favourable survival duration (p=0.006, p<0.001 and p<0.001, respectively). Synchronous visceral metastases adversely affected the survival duration (p=0.008).

**Conclusion:**

Single-stage posterior decompression and stabilisation improved the clinical outcomes of spinal pain and neurological deficit in metastatic spinal tumours. Type of primary tumour, visceral metastasis, perioperative morbidity, and post-operative ambulatory status significantly impact post-operative survival duration.

## Introduction

The spine is one of the most common locations affected when the primary malignancy involves the skeletal system^1,2^. Patients’ function and quality of life are greatly affected, especially in the event when there is the presence of metastatic spinal cord compression that causes neurological deficits^3,4^. These patients eventually develop paraplegia or tetraplegia and sphincteric incontinence if they are left untreated^5^. Acute onset of metastatic epidural spinal cord compression (MSCC) requires urgent treatment, which was reported in up to 14% of cancer patients with spinal metastases^6^. Surgical intervention is indicated for decompression and restoration of neurologic function, and also to address the stability of the unstable spinal column^7^. Patchell *et al* reported a superior clinical outcome with direct surgical decompression in addition to radiotherapy compared to radiation therapy alone from a randomised trial^6^.

The role of surgery is generally considered as a palliative treatment. However, recent literature suggested longer postoperative survival and better clinical outcomes with decompression of the spinal cord with stable, rigid fixation^8,9^. These patients were able to achieve early mobilisation, regain useful ambulation or urinary function, reduce pain, improve quality of life, and prolong survival^10^. Therefore, objectively analysing patients before surgery and during follow-up regarding their clinical outcomes is worthwhile. Hirabayashi *et al* and Chong *et al* reported favourable clinical outcomes in similar studies^11,12^. Limited literature has been published in Malaysia on clinical outcomes of spine metastases requiring surgical intervention. Thus, analysing this group of patients would enhance our knowledge of the multidisciplinary palliative treatment options.

This retrospective study was designed to review our experience with surgical intervention for spinal metastases in a single institution and to analyse the clinical outcomes of the patients in neurological deficit, pain reduction, and survival.

## Materials and Methods

Between June 2015 and June 2022, a total of 63 patients were diagnosed with thoracolumbar spinal metastases and underwent surgical treatments. The medical records of these patients were reviewed retrospectively. Twelve patients were excluded from the study due to insufficient documented data and/or not turning up for follow-up visits. Surgeries done for metastases to the cervical spine were omitted. The remaining 51 patients were recruited and analysed. Ten patients remained alive at the time of review, with a median follow-up period of 30.6 months (range 9–84 months). All patients underwent palliative surgeries with posterior instrumented stabilisation using pedicle screws and indirect decompression for thoracolumbar spine. Indications of surgery were neurological deficit, spinal instability or a combination of both. Spinal instability included mechanical instability when the spine failed to maintain physiological spinal load, and clinical instability resulted in neurological deficit, spinal pain or a combination of the symptoms^13^.

Patients’ demographics collected were the age at the time of surgery, gender, ethnic group, primary site of carcinoma, and evidence of distant metastasis to the viscera and bone in addition to the spine. The locations of spine metastases were subdivided into semi-rigid, mobile and junctional segments^14,15^. The spinal levels from T3-T10 were classified into semi-rigid segments, the levels from L2-L4 were classified into mobile segments, whereas the junctional segment included the levels of T1-T2, T11-L1 and L515. The pre-operative and post-operative clinical assessments collected were the neurological status and pain score. Neurological assessment was documented pre-operative and post-operatively with reference to the American Spinal Injury Association impairment scale (ASIA)^16^. The patient with the neurologic status of ASIA E had normal motor and sensory function. The patient with no motor or sensory function preserved in the sacral segments S4-S5 would be graded ASIA A complete neurological deficit. Those patients with ASIA grades B, C and D suffered from the incomplete motor and sensory loss. Patients in the category of ASIA D and E were able to ambulate, whereas patients diagnosed with ASIA grades A, B and C were considered non-ambulatory. Spinal pain described by the patients were local back pain, radiating pain or both^14^. Pain score was graded using the visual analogue scale (VAS), from zero (no pain) to 10 (maximal pain experienced)^17^. Patients’ survival was defined from the time of surgery to the date of death or last appointment seen during outpatient clinic follow-up. The primary types of cancers were classified into slow, moderate and rapid growth, whereas myeloma and lymphoma were considered separately^18,19^.

The study was approved by the Medical Research and Ethics Committee (MREC), Ministry of Health Malaysia with National Medical Research Registry (NMRR) ID-22-01375-SU6 (IIR).

Data analysis was performed with the SPSS Statistics software package, version 27 [Chicago. IL, USA]. Descriptive statistics of the continuous variables with normal distribution were expressed in mean with standard deviation (SD), whereas median and interquartile range (IQR) were applied for non-normally distributed variables. The median pain score (VAS) was compared using the Mann-Whitney U test, Wilcoxon signed rank test or Kruskal-Wallis test where appropriate. The Chi-Square and Fisher’s Exact tests were used to analyse the ASIA/ambulation status. Patient survival was studied by performing a Kaplan-Meier analysis. The factors that influenced the survival duration were analysed using the log-rank test. Statistical significance was determined with the p<0.05.

## Results

A total of 51 patients were reviewed after inclusion and exclusion criteria were applied (n=51). The mean age was 57.5 years (range 30–85 years) at the time of surgery. Twenty-four (47.1%) male and 27 (52.9%) female patients were included. For ethnic groups, Malay and Chinese had the same number of 23 (45.8%) patients each, whereas Indian consisted of 5 (9.8%) patients. The most common origin of the primary carcinoma was the breast in 12 (23.5%), followed by the lung in 7 (13.7%), colorectal in 5 (9.8%), prostate and kidney in 4 (7.8%) cases each, the bone marrow, nasopharynx and unknown in 3 (5.9%) cases each, thyroid, liver and lymphatic in 2 (3.9%) cases each and other sites in 4 (7.8%) that consisted of the uterus, bladder, cervix and penis one case each. The involvement of the spine was mainly multiple segments that occurred in 39 (76.5%) patients whereas 12 (23.5%) patients were found to have only single-level metastasis. Bony metastases other than axial spine happened in 13 (25.5%) patients, while the remaining 38 patients (74.5%) did not have other bony lesions. Twenty-seven (52.9%) patients had evidence of metastases to visceral organs. The patient demographics are summarised in Table I. All patients with breast cancer had multiple spine metastases, while the patients diagnosed with lung cancer had the highest (85.7%) visceral involvement.

**Table I:** Patient demographic and the extent of metastases.

			Extent of involvement	
		Multiple spine	Other skeletal	Visceral organ
Number of patients, n (%)	51	39 (76.5)	13 (25.5)	27 (52.9)
Gender, n (%)				
Male	24 (47.1)			
Female	27 (52.9)			
Ethnic groups, n (%)				
Malay	23 (45.8)			
Chinese	23 (45.8)			
Indian	5 (9.8)			
Primary carcinoma, n (%)				
Breast	12 (23.5)	12	4	3
Lung	7 (13.7)	4	3	6
Colorectal	5 (9.8)	2	1	3
Prostate	4 (7.8)	4	2	2
Kidney	4 (7.8)	1	2	2
Marrow	3 (5.9)	2	0	2
Nasopharynx	3 (5.9)	3	0	1
Unknown	3 (5.9)	3	0	3
Thyroid	2 (3.9)	2	0	0
Liver	2 (3.9)	1	0	0
Lymphatic	2 (3.9)	2	0	2
Others	4 (7.8)	3	1	3

**Fig. 1:**
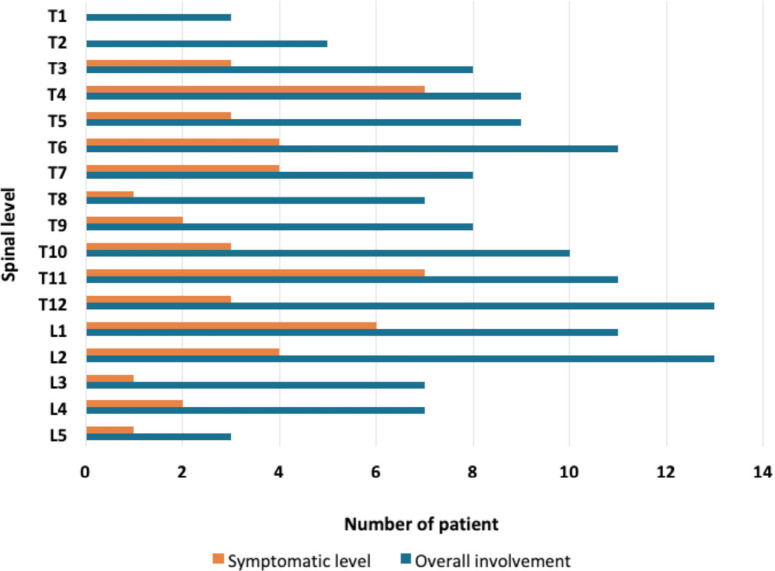
The overall involvement and symptomatic level of thoracolumbar spine.

In thoracolumbar segments, T4 and T11 were the most frequent levels that caused symptoms. Twenty-four (47.1%) symptomatic levels were in the semi-rigid segment, 7 (13.7%) in the mobile segment, and the remaining 20 (39.2%) were in the junctional segment. The extent of spinal levels affected (overall detected from the Magnetic Resonance Imaging) and the symptomatic segments are shown in Fig. 1.

In all the patients, single-stage surgery was performed through a posterior approach with pedicle screws long segment instrumentation for stabilisation, and wide laminectomy for indirect decompression when there was evidence of metastatic epidural compression. The long segment fixation spanned two levels above and two below the affected segment(s). No anterior resection or reconstruction procedure was performed. Transpedicular biopsy was taken concurrently with the surgical procedure.

There was no intra-operative death reported. Two (3.9%) surgical mortalities were observed at 14 days postoperatively. The most common perioperative morbidity noted up to 30 days post-surgical treatment was decubitus sore, which happened in 13 (25.5%) patients. Urinary tract infection occurred in 5 (9.8%) patients, followed by hospital acquired pneumonia in 4 (7.8%), surgical site infection in 2 (3.9%), deep vein thrombosis and acute myocardial infarction in one (1.9%) each. Surgical complications happened in 2 cases (3.9%) with wound dehiscence due to surgical site infection. There was no fixation failure, cerebral spinal fluid leakage or post-operative epidural haematoma.

Twenty (39.2%) patients received chemotherapy either pre-operatively or post-operatively as adjuvant therapy. Postoperatively, 16 (31.4%) patients were subjected to radiotherapy to the spine. A total of 12 (23.5%) patients received both chemotherapy and radiotherapy as adjuvant therapy.

The overall median pre-operative spinal pain VAS was 5 (IQR 5.0-7.0), which reduced significantly post-operatively to 2 (IQR 1.5-3.0) at two weeks follow-up assessment (p<0.001). A further drop of median VAS to one (IQR 0-2.25) was observed at three months (p=0.009). The VAS maintained a low at 0.5 (IQR 0-1.75) at six months follow-up.

Patients with a single spinal segment involvement had higher median VAS of 7 (IQR 5.5-8.0) compared to multiple spinal segment metastases VAS 5 (IQR 5.0-6.0) reported before surgical treatment (p=0.018). During the initial presentation, there was no significant difference in VAS between genders. The median VAS for both males and females were 5 (IQR 5.0-7.0) (p=0.809). Spinal metastases from the thyroid had the highest median VAS of 7.5 (IQR 6.0-9.0), followed by colorectal median VAS of 7 (IQR 7.0-8.0). The lowest VAS in spinal metastases was from the bone marrow, with a median VAS of 4 (IQR 3.0-5.0), and the nasopharynx, with a median VAS of 3 (IQR 3.0-4.0). The origin of the primary tumour did not have a significant difference in VAS (p=0.058). Spinal metastases to the mobile segment had the highest median VAS of 6 (IQR 5.5-8.0), followed by the junctional segment with a median VAS of 5 (IQR 5.0-7.0) and the median VAS for the semi-rigid segment was 5 (IQR 4.5-6.0). The pain score (VAS) is summarised in Table II. The location of metastatic spine lesions (junctional vs mobile vs semi-rigid segments) did not have a significant difference in VAS (p=0.186). The patients who did not receive perioperative adjuvant therapy had higher median VAS of 6 (IQR 5.0-7.0) than those who received perioperative adjuvant therapy. The patients who received perioperative chemotherapy had a median VAS of 5 (IQR 5.0-6.0), the patients who received post-operative adjuvant radiotherapy had a median VAS of 5 (IQR 5.0-5.0), and the patients who received a combination of therapies had a median VAS of 5 (IQR 4.0-6.5). There was no significant difference in the median VAS for patients who did not receive adjuvant therapy and the types of peri-operative adjuvant therapy (p=0.53).

**Table II:** Pain score after surgical treatment.

	Visual Analog Scale	
	Pre-operative	Post-operative
Overall, median (IQR)	5.0 (5.0-7.0)	2.0 (1.5-3.0)
Primary carcinoma, median (IQR)		
Breast	5.0 (3.5-6.0)	2.0 (2.0-2.0)
Lung	6.0 (5.5-7.0)	2.0 (2.0-3.5)
Colorectal	7.0 (7.0-8.0)	2.5 (2.0-3.0)
Prostate	5.0 (5.0-5.5)	3.5 (2.0-5.0)
Kidney	6.0 (4.0-7.5)	1.0 (1.0-1.0)
Marrow	4.0 (3.0-5.0)	3.0 (2.0-3.0)
Nasopharynx	3.0 (3.0-4.0)	1.0 (1.0-2.0)
Unknown	6.0 (4.5-7.0)	0.5 (0-1.0)
Thyroid	7.5 (6.0-9.0)	3.5 (2.0-5.0)
Liver	5.0 (5.0-5.0)	2.0 (1.0-2.0)
Lymphatic	6.0 (5.0-7.0)	0.5 (0-1.0)
Others	5.0 (4.0-6.0)	1.0 (0-2.0)
Spine segment, median (IQR)		
Semi-rigid (T3-T10)	5.0 (4.5-6.0)	2.0 (2.0-3.0)
Mobile (L2-L4)	6.0 (5.5-8.0)	4.0 (2.5-5.0)
Junctional (T1-T2, T11-L1, L5)	5.0 (5.0-7.0)	1.5 (0.5-2.0)

Abbreviation - IQR: interquartile range

Neurological results showed 15 (29.4%) patients presented with ASIA A, 6 (11.8%) patients with ASIA B, and 10 (19.6%) patients each for ASIA C, ASIA D and ASIA E. The average time of onset of neurological deficits for ASIA A patients was 6.4 (range 1–30) days. Post-operatively 11 patients with neurological deficit ASIA A remain unchanged; 2 of them improved to ASIA C, and another 2 of them improved to ASIA D. The average duration from admission to time of surgery was 4.1 (range 1–8) days. The changes in the neurologic status are summarised in Table III. Thirty-one (60.8%) patients’ neurological status remained unchanged, 18 (35.3%) patients reported improved neurology whereas 2 (3.9%) patients had worsened neurological deficits. Improved ASIA grade was observed in 3 (25%) patients diagnosed with breast cancer, 2 (28.6%) in lung cancer, 2 (40%) in colorectal cancer, 2 (50%) in prostate and kidney cancer each, 2 (66.7%) in myeloma and nasopharyngeal cancer each, and one (50%) in thyroid and liver cancer each. Eight (15.7%) of the patients improved from the non-ambulatory group to become ambulatory (p<0.001). A higher percentage of improved ASIA grade was observed in single-level spinal metastasis compared to multiple-level involvement (41.7% vs 33.3%). Combined chemotherapy and radiotherapy reported better improvement in ASIA grade (58.3%). However, neither the primary type of carcinoma, the number of spine segment involvement, nor the type of adjuvant therapy had a significant number of patients who gained ambulation (p=0.893, p=0.178 and p=0.351, respectively).

**Table III:** Neurological status after surgical treatment.

		Post-operative ASIA grade		
		Improved (Gained ambulation)	Unchanged	Worsened
Number of patients, n	51	18 (8)	31	2
Primary carcinoma, n				
Breast	12	3 (2)	9	-
Lung	7	2 (1)	5	-
Colorectal	5	2 (1)	2	1
Prostate	4	2 (1)	2	-
Kidney	4	2	2	-
Marrow	3	2 (1)	1	-
Nasopharynx	3	2 (1)	1	-
Unknown	3	-	2	1
Thyroid	2	1 (1)	1	-
Liver	2	1	1	-
Lymphatic	2	-	2	-
Others	4	-	4	-
No. of spinal metastasis, n				
Single	12	5 (2)	6	1
Multiple	39	13 (6)	25	1
Adjuvant therapy, n				
No therapy	27	7 (3)	19	1
Chemotherapy	8	2 (1)	6	-
Radiotherapy	4	2	2	-
Combination therapy	12	7 (4)	4	1

Abbreviation - ASIA: American Spinal Injury Association

**Fig. 2:**
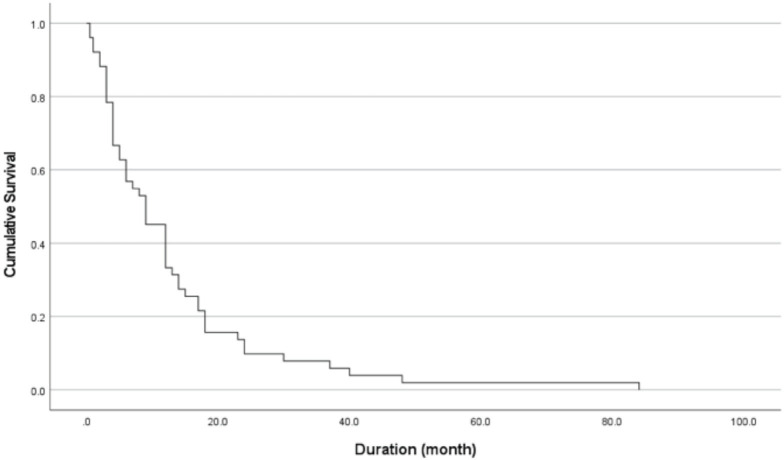
Overall survival curve of patients with median duration of nine months.

The median overall survival duration for the 51 patients was nine months (range 0.5–84 months). The Kaplan-Meier survival curve for the overall patients is shown in Fig. 2. Thyroid carcinoma recorded the highest median survival duration, 31.5 (range 15–48) months. It was followed by myeloma 18 (range 1–84) months, lymphoma 15 (range 7– 23) months, breast carcinoma 12 (range 3–40) months, colorectal carcinoma 9 (range 3–12) months, prostate carcinoma 9 (range 3–37) months, liver carcinoma 7 (range 5–9) months and lung carcinoma 6 (range 2–24) months. The lowest median survival was observed in nasopharyngeal carcinoma, unknown primary, and others, which ranged from 3 to 4 months. Perioperative mortality was reported in one case of endometrial carcinoma and one case of unknown primary. Both of these two patients died two weeks after their surgeries.

A few factors were identified to have significantly prolonged the duration of patient survival, as summarised in Table IV. Spinal metastases from breast, thyroid and prostate carcinoma were classified into the slow-growth group. The other primary carcinomas were classified into moderate and rapid growth groups. Myeloma and lymphoma were considered separately. The slow growth group had a median survival of 12.5 (range 3–48) months, better survival compared to moderate and rapid growth tumour groups with a median survival of 5 (range 0.5–24) months (p=0.006). There was no significant difference in survival within the slow growth group between thyroid, breast and prostate carcinomas (p=0.331, p=0.198 and p=0.377, respectively) compared to myeloma and lymphoma (p=0302 and p=0.805, respectively).

**Table IV:** Analysis of factors associated with post-operative survival.

Factors	P-value
Gender	
Male vs Female	0.06
Ethnic group	
Malay vs Chinese	0.224
Malay vs Indian	0.615
Chinese vs Indian	0.045
Primary carcinoma	
Slow growth vs moderate and rapid growth	0.006
Slow growth vs myeloma	0.302
Slow growth vs lymphoma	0.805
Synchronous distant metastasis	
Visceral metastasis (absent vs present)	0.008
Spinal segment metastasis (single vs multiple)	0.452
Extra-spinal bone metastasis (absent vs present)	0.663
Adjuvant therapy	
No therapy vs Chemotherapy	0.127
No therapy vs Radiotherapy	0.155
No therapy vs Combine Chemotherapy and Radiotherapy	0.241
Perioperative morbidity	
With vs Without	<0.001
Neurological status	
Pre-operative ambulatory vs Non-ambulatory	0.009
Post-operative ambulatory vs Non-ambulatory	<0.001

The median survival for male patients was 6 (range 0.5–84) months and 12 (range 0.5–48) months for female patients. There was no significant difference in survival based on gender (p=0.06). The median survival for Malay was 12 (range 0.5–84) months, Chinese 6 (range 1–37) months and Indian 18 (0.5–48) months. The median survival of patients without visceral metastases was 12 (range 0.5–48) months, and those with visceral metastases had 4 (range 0.5–84) months survival. The survival duration was significantly affected when there were detectable visceral metastases (p=0.008). The median survival rate of patients with metastases to bone (excluding spine) was 6 (range 3–40) months, and those without bone metastases had 9 (range 0.5–84) months of survival. The median survival for the patients with a single spinal metastasis was 12 (range 3–24) months, while patients with multiple spinal metastases had 9 (range 0.5–84) months survival. Metastases to other bone or multiple-level spine involvement did not influence the survival (p=0.663 and p=0.452, respectively).

The pre-operative median survival for ambulatory patients was 12.5 (range 0.5–37) months, better compared to non-ambulatory patients’ median survival of 4 (range 0.5–84) months (p=0.009). Post-operatively, the median survival for ambulatory patients was 13 (range 0.5–84) months, which was significantly longer compared to non-ambulatory patients’ median survival of 4 (range 0.5–24) months (p<0.001). The median survival for patients without perioperative morbidity was 12 (range 0.5–84) months, better compared to patients with perioperative morbidity with a median survival of 4 (range 0.5–24) months (p<0.001). The median survival of the patients without perioperative adjuvant therapy, with chemotherapy, with radiotherapy and with a combination of therapies were 6 (range 0.5–48) months, 13.5 (range 4–84) months, 14 (range 12–37) months and 12 (range 3–40) months, respectively. There was no statistical difference in patient survival (p=0.127, p=0.155 and p=0.241, respectively).

## Discussion

There were variations in the demographic of patients in various series. Lung cancer was reported as the highest percentage of operated spinal metastases cases in earlier similar series^12,14^. A higher percentage of male gender was reported as opposed to our series, which consisted of more females with higher incidences of breast cancer. These factors contributed to limitations in the interpretation of survival based on gender.

The ethnic groups of our patients comprised of equal distribution for Malay and Chinese (45.8%), while Indian was 9.8%. This finding was not proportionate to our population, which comprised of 55.6% Malay, 23.4% Chinese, and 7.0% Indian ^20^. The discussion about this aspect was scarce in the literature. Malaysia National Cancer Registry found that Chinese had the highest overall cancer incidence. It was reported that the age-standardised incidence rate (ASR) for Chinese males was 106.1 and 117.3 for Chinese females. The ASR for Malay males was 73.6 and 90.9 for Malay females. Indian males recorded ASR of 66.7 and 106.9 for Indian females^20^. In our study, Indians had the longest post-operative survival, whereas Chinese had the shortest survival duration. However, the small number of Indian patients with only five types of primary tumour histologies (thyroid, lung, prostate, bone marrow and endometrium) did not represent the overall survival.

In our series, the distribution of spine segment involvements was more at the upper thoracic and thoracolumbar junctions as reported similarly in the literature^14^. Surgical mortality within 30 days was reported from 3% to 4%^12,21^. We noted 2 (3.9%) cases that patients died within 2 weeks. However, their causes of death were not directly due to the surgical sequelae.

The median pre-operative VAS dropped from 5 to 2 following rigid spinal instrumentation in our study, similar to the magnitude of improvement in the other studies^14,19,22^. The significant improvement in pain control was evident during immediate post-operative assessment and further follow-up at three-month duration. Overall, adequate pain relief was achieved in the literature for 68-90% of the patients^12,23,24^. Hence, surgery was a worthwhile option to control pain when non-surgical measures failed.

The gain of useful neurological function from non-ambulatory pre-operatively to ambulatory post-operatively was reported 18 - 59% in the literature^7,12,14,24^. There was 8 (25.8%) patients who gained ambulation from the 31 non-ambulatory patients before surgery in our study. A higher percentage of total neurological deficit (ASIA A) patients (29.4%) in this current series was one of the limiting factors. The possible reasons were to various patient factors or delayed referral to our centre. In Malaysia it was found that the majority of cancers were diagnosed at Stage III (22.8%) and Stage IV (40.9%)^20^. Post-operatively, when a patient maintained or improved in the ambulatory function, it was likely to remain till late in the survival interval^12^. Eighteen (35.3%) patients had improved at least one ASIA grade, similarly, reported in the literature (32-49%)^12,14^. Immediate deterioration of neurological status post-operatively was reported up to 5.7%^14^. Two cases of deteriorated neurological ASIA grade were noted in our study. The first case was a patient with sigmoid colon carcinoma who was able to walk but suffered from disease progression at six months postoperatively with a progressive drop of lower limb power to ASIA B. Another patient with unknown primary had one grade ASIA drop from grade C to grade B during a one-month follow-up with a concomitant poor general health condition.

In the literature, the patients with better pre-operative Karnofsky Performance scores (KPS) and neurological status had a higher possibility of maintaining ambulatory function post-operatively^14,23,25^. Unfortunately, KPS was not routinely documented in our data. In our study, we noticed that patients with single-segment spine involvement and perioperative combined adjuvant therapy had a higher percentage of post-operative improvement of ASIA grade. However, this finding was not statistically significant as described in a similar study^24^.

The median post-operative survival in our series was nine months, and this finding was in line with previous similar studies^12,23^. This finding served as a good guideline and justification for surgical indication. A meta-analysis conducted by Klimo *et al* pointed out that the average one-year survival rate was 41% (range 12–62%) for patients who underwent surgical treatment for metastatic spine disease^7^. We achieved a one-year survival rate of 34.1%. The longest survival time was reported in patients with the primary tumour from the bone marrow, thyroid and prostate^12^. Similar findings were observed in our series for cases diagnosed with thyroid carcinoma and myeloma. The primary site of carcinoma has been noted to influence survival^8,12,21,26^. The primary tumour type, which was classified into the slow growth group, had a significantly longer survival in this study. Distant metastases to visceral organs served as a poor prognostic factor for survival in literature^21,26^. In our study, visceral metastasis had adversely affected patient survival.

In a similar study, Chong *et al* pointed out that post-operative adjuvant therapy strongly prognosticated patients’ survival^12^. In contrast, perioperative adjuvant therapy, both chemotherapy and radiotherapy did not significantly influence the survival duration in our study. However, this observation had to be interpreted cautiously due to the lack of oncology facilities during the earlier years. Nevertheless, this limitation has improved gradually over the years in our institution.

The post-operative ability to ambulate was reported by Hirabayashi *et al* as a significant factor that had prolonged survival, in addition to preventing bedridden complications^11^. We found that these factors were also reflected in our series. Pre-operative ambulatory status was also reported to have affected survival in the literature, although some authors reported otherwise^10,12,27^. In our series, we found that the presenting ambulatory status influenced the duration of postoperative survival (p=0.009).

This study was limited by the small sample size and retrospective nature of data collection within a single institution. A local multicentre prospective randomised study would allow for further subgroup analysis and broader applicability. The quality-of-life assessment and documentation needed to be improved in our record; hence, this essential aspect could not be interpreted.

## Conclusion

Single-stage posterior decompression and stabilisation improved the clinical outcomes of spinal pain and neurological deficit in metastatic spinal tumours. The type of primary tumour, visceral metastasis, perioperative morbidity and post-operative ambulatory status significantly impact the patient survival.
